# Prevention and Treatment of Atrioesophageal Fistula Related to Catheter Ablation for Atrial Fibrillation

**DOI:** 10.19102/icrm.2019.100505

**Published:** 2019-05-15

**Authors:** George M. Bodziock, Caleb A. Norton, Jay A. Montgomery

**Affiliations:** ^1^Department of Internal Medicine, Vanderbilt University Medical Center, Nashville, TN, USA; ^2^Division of Cardiovascular Medicine, Vanderbilt University Medical Center, Nashville, TN, USA

**Keywords:** Ablation, atrial fibrillation, atrial fibrillation ablation, atrioesophageal fistula, complications

## Abstract

Atrioesophageal fistula (AEF) is an uncommon but devastating complication of catheter ablation for atrial fibrillation. Even with appropriate recognition and treatment, mortality is greater than 30% in most studies. If AEF is suspected, it is essential to avoid endoscopy and to order immediate cross-sectional imaging. If the diagnosis is confirmed, a thoracic surgeon should be promptly notified and must assess the patient urgently. The prognosis for AEF is poor even if it is appropriately recognized and addressed, so prevention must be a high priority. Prevention of AEF should involve the use of low-risk and cost-effective measures during ablation, which may increase safety, efficacy, or both. These strategies may include conscious sedation (as opposed to general anesthesia), low-power ablation, low-flow irrigation, short-duration lesions, esophageal temperature measurement, esophageal deviation, and pharmacologic prophylaxis with proton pump inhibitors or histamine H_2_ receptor blockers. Multiple new technologies are now becoming available, which may further reduce esophageal injury. Proceduralists should be aware of the available techniques and equipment that may help to reduce the risk of AEF, while simultaneously considering the possibility of unintended consequences.

## Introduction

Atrial fibrillation (AF) is the most common clinically relevant arrhythmia in the world, with more than five million new cases presenting per year.^1^ This number is expected to grow as the population ages, as the prevalence of AF in those older than 80 years is almost 10%.^2^ In the United States, about 50,000 catheter ablation procedures for AF are performed annually.^1^ In recent reports, the rate of major complications from AF ablation is less than 5%.^1,3^ These complications chiefly consist of vascular events such as hemorrhage, myocardial infarction, stroke, cardiac tamponade, and atrioesophageal fistula (AEF).^4^ The last is the least common, occurring in less than 0.1% of cases, but it can have delayed and devastating consequences.^1,3,5^ AEF can be rapidly fatal, even with appropriate treatment.^1^ It is the second most common cause of mortality related to AF ablation and is linked to 16% of postprocedural deaths.^2^ Because of this, the risk of AEF informs and constrains the method of ablation on the posterior wall of the left atrium (LA) in an attempt to prevent this complication. In this review, we will discuss appropriate safety measures as well as new and experimental techniques and equipment aimed at preventing AEF while obtaining durable lesion formation on the LA posterior wall. We will also discuss optimal management of AEF and associated conditions.

## Pathophysiology

During catheter ablation for AF, esophageal heating may occur as a result of the proximity of the esophagus to the posterior wall of the LA.^2,5^ The distance between the sites of endocardial ablation and the esophagus significantly predicts esophageal temperature rise during AF ablation.^6^ This relationship varies among patients, and the amount of connective tissue between the LA and esophagus may differ.^2,5^ The position can even be variable in a single patient, as the esophagus can shift by more than 4 cm during ablation.^2,5^ Although the exact mechanism of AEF is controversial, the likely route of injury is direct thermal injury due to the close proximity of the LA to the esophagus.^2,5^ Other possibilities include the exacerbation of acid reflux due to damaged vagal nerve branches and ischemia resulting from the cauterization of end arterioles.^5^ Interestingly, the LA is relatively spared from direct necrosis or late perforation from ablation, with no known reports of this injury to the LA alone.^5^ Rather, esophageal injury with ulcer formation can lead to erosion and fistula formation from the esophagus towards the LA, ultimately penetrating the LA in some cases.^1,2,5^ The esophageal lesion may communicate with the pericardium first or alone, forming an isolated atriopericardial fistula, depending on the patient’s anatomy and area of injury.^5^ If the communication continues through the atrial wall, an AEF results.^5^ This can lead to air and food extravasation into the LA, causing infections, embolism, and other catastrophic complications.^5^

## Clinical presentation

Patients typically present with symptoms at between two weeks and six weeks after the procedure, although they can present even earlier or later in some cases.^1,5^ Symptoms are usually nonspecific but can include chest discomfort, nausea and vomiting, dysphagia, odynophagia, fever, and malaise.^5^ Hematemesis and melena are less common, as the communication usually acts as a one-way valve from the esophagus to the atrium.^5^ Clinicians should be concerned about AEF when a patient presents with a sudden onset of chest pain followed by constitutional symptoms after AF ablation.^5,7^ The most common presenting symptoms in a review of 53 reported cases were fever (44%), neurological deficits (27%), hematemesis (19%), altered mental status (15%), and chest pain (11%).^5,7^ Regardless of the presenting symptom, however, patients can progress to develop upper gastrointestinal bleeding, pleural and/or pericardial effusions, mediastinitis, sepsis, and embolic strokes.^2^

## Diagnosis

If there is clinical suspicion for AEF, upper endoscopy (EGD) should not be performed due to the risk of systemic air embolization and stroke from air insufflation during EGD.^2^ When EGD was performed in cases of suspected AEF, 28% of patients had rapid deterioration, including death, after the EGD.^1^ Instead, cross-sectional imaging should be obtained. The most widely recommended modality is thoracic computed tomography (CT) scan with oral and intravenous contrast; however, magnetic resonance imaging (MRI) of the chest can also be considered, depending on institutional expertise.^5^ Contrast-enhanced CT scan of the chest reveals abnormal findings in 98% of cases.^1^ Physicians should look for signs of pericardial effusion, atrial and esophageal inflammation, intravascular air, frank communication between the esophagus and the atrium or pericardium, and evidence of septic or food emboli.^2^ In addition, CT or MRI of the head (to look for multifocal air embolism) should be considered. Routine laboratory assessment including white blood cell count and blood cultures should be performed.^5,7^

## Treatment

The management of AEF occurring after AF ablation begins with the recognition of this rare yet potentially life-threatening complication. Given the relatively low incidence, management is largely based on case reports and case series. However, these available reports strongly suggest that AEF should be treated as an interventional emergency requiring prompt surgical evaluation. Although simultaneous treatment with antibiotics and appropriate medical support are vital, expectant medical management alone has been associated with increased mortality and poor outcomes.^1,2^ Surgical intervention is therefore the standard of care and, if AEF is suspected, thoracic surgery consultation should be obtained immediately, keeping in mind that most physicians will not have extensive clinical experience with this entity.^8^

In a systematic review, Singh et al. found 16 cases of AEF primarily managed with surgical intervention and seven cases managed with esophageal stenting. All seven cases managed with esophageal stenting had fatal outcomes. Additionally, of the 16 cases managed via surgical intervention, seven patients died (44%).^9^ They also described the clinical outcomes of six previously unpublished cases of AEF following AF ablation in a multicenter series.^9^ In five of the six cases, the repair was performed during cardiopulmonary bypass with pericardial patch repair of the posterior wall of the LA via an atriotomy.^9^ The remaining patient received pledgeted suture repair of the LA, which did not require bypass.^9^ Primary repair of the esophagus via intercostal muscle flap or doubled-over pericardium was performed in four of the six cases but not in the two remaining cases due to the esophagus not being well-visualized.^9^ Various other case reports in the literature have described the successful management of AEF utilizing transthoracic extracardiac repair without cardiopulmonary bypass.^10,11^ In a separate case report, St. Julien et al. discussed their successful treatment of AEF via a novel technique of esophageal ligation and decompression by a cervical approach, with the avoidance of cardiopulmonary bypass.^12^

Although the management of AEF following catheter-based AF ablation seems to be best done primarily via surgical intervention, there are a number of case reports of the successful management of esophageal perforation without atrial communication utilizing minimally invasive techniques. Bunch et al. first described the successful management of esophageal perforation without AEF after AF ablation utilizing esophageal stenting.^13^ Ellis et al. also described a case of esophageal perforation after AF ablation that was successfully managed with esophageal stent placement.^14^ In both instances, the stents were placed temporarily for three weeks and four weeks, respectively, and the esophageal perforation did not progress to AEF. Both patients underwent a repeat CT chest scan to confirm the healing of the esophageal perforation, in addition to a barium swallow study before stent removal.^14^

Eitel et al. reported a series of three patients who developed esophagopericardial fistula and were successfully managed with esophageal stenting and pericardial drain placement. Of note, one patient in this case series underwent EGD prior to pericardial drain placement and subsequently developed pneumopericardium. Thus, the authors suggested that pericardial drain placement be completed before EGD in cases of suspected esophagopericardial fistula.^15^ As mentioned above, EGD is contraindicated in cases where AEF is suspected due to the risk of air embolism, which can ultimately lead to death.^15^

## Prognosis

Although there are limited data available for this rare complication, most reports suggest that mortality in AEF exceeds 50% without treatment, with the worst outcomes seen in patients presenting with gastrointestinal bleeding and neurologic symptoms.^1^ Even with prompt recognition and optimal treatment, mortality is still reportedly around 33%.^1^ The time interval from ablation to the development of AEF does not predict mortality.^5^ Delayed recognition or the use of conservative treatment strategies a much higher associated mortality rate.^1^

## Prevention

Various strategies can be employed to reduce the risk of AEF **(Table 1)**. Given the rarity of AEF, preventive measures should be low-risk and relatively inexpensive or should concomitantly improve outcomes of AF ablation. Factors that may affect the risk of esophageal injury and AEF formation include the type of sedation, ablation modality and power settings, esophageal temperature monitoring, esophageal deviation, and pharmacologic prophylaxis.^1,5^

### Sedation

Conscious sedation (as opposed to general anesthesia) seems to be protective and likely reduces esophageal contact.^2,5,16^ In a study by Di Biase et al. involving 50 patients undergoing ablation, 25 were randomized to general anesthesia and 25 were randomized to conscious sedation with fentanyl or midazolam. Esophageal tissue damage as visualized by capsule endoscopy on the first postoperative day was seen in just one patient under conscious sedation and 12 patients under general anesthesia, but none of the patients had a major complication related to the visualized lesions.^16^ Patients under general anesthesia had higher maximum lower esophageal temperatures.^16^ Several factors may account for this, including reduced esophageal motility with general anesthesia and the patients’ inability to alert the operator of any discomfort.^2,5,16^ Additionally, a lack of swallowing may contribute to the fixation of esophageal positioning.^2,5,16^

### Ablation modality and power

AEF has been observed most frequently with radiofrequency ablation as compared with other ablation modalities, although it is possible that this is due to the higher overall number of procedures using this technique. Small studies,^17–19^ have shown that lower radiofrequency energy used during ablation reduces esophageal injury, and many operators thus apply no more than 25 W to 30 W of energy on the posterior LA wall based on these findings.^2^ As opposed to nonirrigated ablation, irrigating the catheter tip with open saline flow to cool the interface has been shown to prevent coagulum formation, reduce the risk of emboli, and enable formation of deeper lesions in the LA wall.^20^ However, irrigating with a lower flow rate (2 mL/min versus 17 mL/min) has been shown to maximize the endocardial lesion diameter while also allowing for reduced deep tissue heating.^20^ Recently, high-power, short-duration irrigated lesions utilizing primarily resistive heating have been described as another alternative to standard ablation techniques, which rely on both resistive and conductive heating for lesion formation.^21^ Leshem et al. demonstrated in a swine model that ablation with the QDOT-Micro catheter (Biosense-Webster, Diamond Bar, CA, USA) at 90 W for four seconds resulted in contiguous transmural lines, as compared with 25 W for 20 seconds, which resulted in gaps and nontransmurality. It remains to be seen whether very-short-duration lesions relying on primarily resistive heating may offer a more predictable lesion depth, which could lead to improved lesion durability and esophageal safety. A clinical trial of this catheter and power delivery (90 W for four seconds) for AF ablation is currently being performed in Europe. Although much of the discussion on AEF prevention has centered around radiofrequency ablation, AEF has been observed after virtually all methods of AF ablation, including surgical ablation, high-intensity ultrasound ablation, and balloon cryoablation, in which AEF most often develops adjacent to the left inferior pulmonary vein (PV).^2,5^

### Temperature monitoring

Direct temperature measurement using a lower esophageal temperature probe is another viable method to reduce esophageal injury, allowing for temperature feedback in real-time, which can be used to titrate energy delivery during LA ablation.^2^ Multiple studies have shown this method to be effective at reducing damage, although it does not eliminate the potential for injury.^2^ This technique also has the possibility of introducing other problems. Uninsulated temperature probes may actually cause injury by acting as electrical conductors, transferring thermal energy directly to the esophagus.^2,5,22^ Using insulated thermocouples should reduce this risk.

There are also new technologies on the horizon, which may offer additional safety features. Early studies suggest that multisensor probes present superior temperature monitoring and may reduce esophageal injury.^23^ One such device is the Circa S-Cath Temperature Monitor (Circa Scientific, Englewood, CO, USA), a flexible probe that conforms to the esophageal shape and which is insulated to reduce conductivity.^24^ It features 12 temperature sensors that provide feedback continuously.^24^ In a recent study, this probe was able to detect 0.2°C temperature fluctuations up to 17 seconds faster than comparable single-sensor probes (mean detection time: 13.4 seconds versus 30.5 seconds).^23,24^

Infrared thermography is another new tool for esophageal temperature management, in which esophageal temperature is measured continuously by an infrared scan rather than direct conduction to a probe.^22^ Daly et al. recently described a study of 16 patients undergoing AF ablation with an infrared probe in place. The proceduralists were blinded to temperature data during the procedures, but standard precautions were used including a reduced ablation energy level of 20 W during ablation of the posterior LA wall.^22^ This study revealed dramatically higher peak temperatures than those typically found with traditional thermal probes, often to a point of over 40°C and occasionally over 50°C, likely due to rapid local temperature fluctuations of short duration that are difficult to measure with a standard conductive temperature probe.^22^ A full EGD was performed on all patients the day after ablation, and only two patients demonstrated thermal lesions in the esophagus.^22^ Both of these patients had temperature peaks of more than 50°C during their procedures.^22^ Given these findings, infrared thermography may provide a viable and possibly superior measurement in comparison with standard probes, although additional investigation is needed to determine the applicability of the findings and outcomes.

### Mechanical esophageal deviation

**Figure 1:**
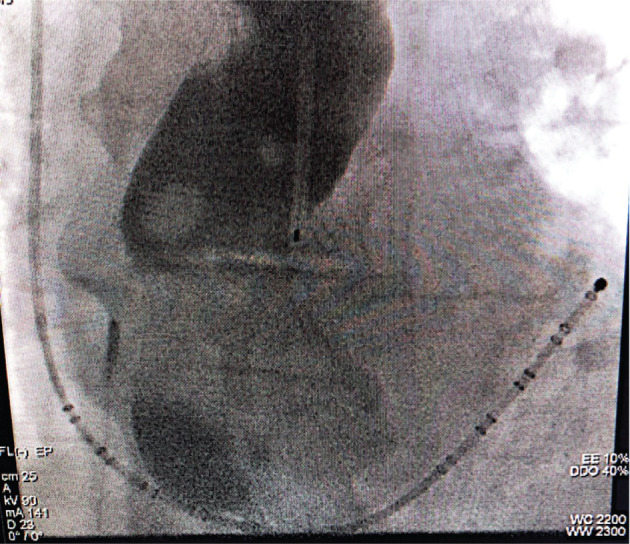
Fluoroscopic imaging showing a linear temperature probe with a barium esophagram. The linear probe alone offers poor delineation of esophageal position. Image used with permission from Dr. Andrea Natale.

Mechanical esophageal deviation with visualization is another strategy that could potentially reduce the risk of AEF. The distance between sites of ablation in the LA and the esophagus reliably correlates with the esophageal temperature rise.^6^ Standard temperature probes cannot be used to deviate the esophagus away from ablation energy and may offer poor estimation of esophageal positioning. In the case of a larger esophagus, the temperature probe may lie relatively distant from portions of the esophagus and closest to the LA posterior wall ablation site **(Figure 1)**. Effective esophageal deviation was described using a deflectable transesophageal echocardiography probe in 2015.^25^ In 2012 and 2017, Koruth et al. presented two studies using a malleable metal stylet inside a plastic tube within the esophagus. In the first 19-patient cohort, the temperature monitor recorded no esophageal temperatures of more than 40°C during the procedures.^26^ Furthermore, postprocedural EGD revealed no esophageal ulceration in this group of patients, except for in one patient who had a congenital esophageal diverticulum across the posterior LA.^26^ It should be noted that EGD did reveal frequent minor esophageal trauma from the manipulation itself, and patients frequently reported a transient “sore throat” afterward.^26^ In a subsequent study in 2017, the same group described deviation in an additional 114 patients, revealing that significantly lower-temperature elevations were achieved with the performance of esophageal deviation than without it. Notably, results were better with leftward deviation during right PV ablation than rightward deviation during left PV ablation, likely due to thoracoabdominal variations.^27^ The achievable deviation distance was also important, with deviations of 20 mm or more from the baseline position having very infrequent temperature elevations as compared with smaller deviations.^27^

**Figure 2:**
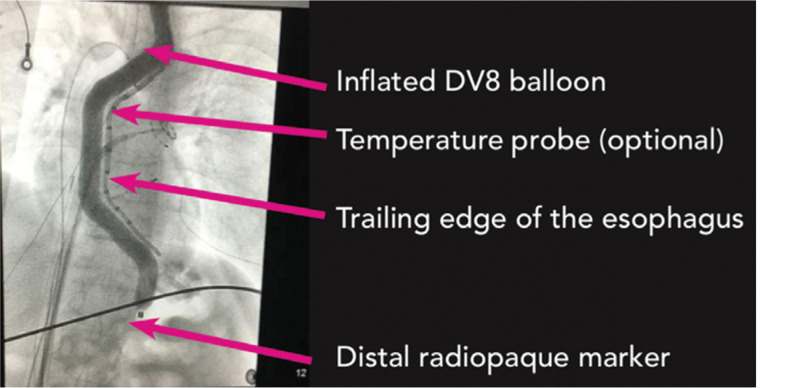
Fluoroscopic imaging showing an inflated DV8 balloon (Manual Surgical Sciences, Minneapolis, MN, USA). Image used with permission from Manual Surgical Sciences.

There are now two devices designed for esophageal deviation available for use in the United States. The DV8 inflatable balloon retractor (Manual Surgical Sciences, Minneapolis, MN, USA) is a novel device that can be deployed in the esophagus and used for deviation during posterior wall ablation **(Figure 2)**. Bhardwaj et al. reported their experience using this device for esophageal deviation in 200 patients undergoing AF ablation, with the average deviation being 21.2 mm ± 8.7 mm during right PV isolation and 15.5 mm ± 6.8 mm during left PV isolation.^28^ Esophageal temperatures of more than 38°C occurred in 100% of patients when deviation was less than 5 mm, 28% of the time when deviation was between 5 mm and 20 mm, and 1.9% of the time when deviation was more than 20 mm.^28^ There were no significant complications noted during their study, including no AEF or signs of esophageal trauma, but two patients did experience transient oropharyngeal bleeding due to trauma related to device placement.

The EsoSure Retractor (Esosure, Boynton Beach, FL, USA) uses a preshaped nitinol stylet that is pliable at room temperature but assumes a more rigid S-shaped frame at body temperature to deviate the esophagus away from the area of ablation. A recent study revealed an esophageal temperature rise of more than 1°C in only 3% of patients using this device versus in 79.4% of 478 propensity-matched controls.^29^ The mean trailing-edge deviation in this study was 2.45 cm (range: 1–4.5 cm).^29^

It is important to keep in mind that any probe in the esophagus, whether being used for temperature monitoring or deviation, may exert an anterior force against the anterior esophageal tissue, moving this tissue toward the LA posterior wall. This effect is likely to be greater for larger and more rigid devices. Therefore, any deviation device should be used judiciously and actively managed, and temperature monitors should be small in the anteroposterior profile. The effects of unintended consequences should be considered and anticipated.

### Pharmacologic prophylaxis

The use of proton pump inhibitors or histamine H_2_ receptor blockers for gastric acid suppression is another preventive measure that may reduce the risk of injury, but it is difficult to provide strong evidence given the rarity of esophageal complications at this time.^2,5^ Multiple regimens have been described, including initiation at five or more days preprocedure (to allow for maximal clinical effectiveness) and continuation for up to six weeks postprocedure. Since this is a relatively low-risk strategy with a low associated cost, it is a reasonable strategy to use empirically following ablation including of the posterior wall of the LA.

### Empiric endoscopy

Empiric endoscopy after posterior LA ablation has been described as a method to screen for injury. Multiple studies have suggested that postablation endoscopy may help to identify patients at higher risk for complications such as AEF, based on the presence of postablation ulcerations.^3,5^ However, implementing this in all affected patients involves increases in costs, anesthesia risk, procedural risk, and time. Discovered injuries must also trigger an effective treatment to make screening viable, and this would certainly consist of gastric acid suppression, which is typically implemented regardless of evidence of esophageal injury. In extreme circumstances, parenteral feedings and temporary esophageal stenting have been pursued, but it is not entirely clear as to what the outcome would have been for these patients if the lesions were not identified. For now, the appropriate standard of care likely includes empiric gastric acid suppression after all ablations of the posterior LA. Postprocedural EGD was found to be reasonable as part of clinical studies and might be considered after perceived high-risk cases, but, in our opinion, it is probably not appropriate as a routine part of postprocedural monitoring in the nonresearch setting. Also of note, if there is any suspicion of AEF formation after ablation, endoscopy must be avoided, as such can lead to immediate complications and even death, as mentioned above.^1^

## Discharge instructions

Constitutional symptoms after AF ablation, especially in association with chest pain, should warrant workup for AEF. Patients should be counseled in writing to notify the AF ablation treatment team if symptoms suggestive of AEF appear within the first 60 days after operation. These include fever, chest discomfort, dysphagia, hematemesis, melena, neurologic symptoms, and dyspnea. Emergency or primary care providers may be unaware of the possibility of AEF and are likely not familiar with the symptoms that warrant a workup for the condition.

## Summary

AEF is an uncommon but devastating complication of catheter ablation for AF.^1–3^ Even with appropriate recognition and treatment, mortality is greater than 30% in most studies.^1–3^ If AEF is suspected, it is essential to avoid endoscopy and order immediate cross-sectional imaging.^1^ If the diagnosis is confirmed, a thoracic surgeon should be promptly notified and must assess the patient urgently.^10,13^ Even if appropriately recognized and treated, the prognosis for AEF is poor, and prevention must be a high priority. Prevention of AEF should involve low-risk and cost-effective measures during ablation, which may increase safety, efficacy, or both. These strategies may include conscious sedation (as opposed to general anesthesia), low-power ablation, low-flow irrigation, short-duration lesions, esophageal temperature measurement, esophageal deviation, and pharmacologic prophylaxis with proton pump inhibitors or histamine H_2_ receptor blockers.^1,2,5^ Multiple new technologies are now becoming available that may further reduce esophageal injury. Proceduralists should be aware of both current and emerging techniques and equipment that may reduce the risk of AEF while also considering the possibility of unintended consequences.^6,22–24,27^

## Figures and Tables

**Table 1:** Categories of AEF Prevention with Potential Strategies and Novel Approaches, Based on the Best Available Evidence

Preventive Category	Potential Strategies and Novel Approaches
Procedural sedation	• Conscious sedation appears superior to general anesthesia
Ablation power settings and irrigation	• Low energy (no more than 25–30 W) • Irrigated catheter tip • Low-flow irrigation (2 mL/min) • Higher-power, short-duration lesions (resistive heating)
Esophageal temperature monitoring	• Insulated thermocouples • Multisensor probes • Infrared thermography
Mechanical esophageal deviation	• DV8 inflatable balloon retractor* • EsoSure retractor**
Pharmacologic prophylaxis (2–6 weeks)	• H_2_ blockers (eg, ranitidine, famotidine, cimetidine) • PPIs (eg, pantoprazole, omeprazole, lansoprazole)

H_2_: histamine H_2_ receptor; PPI: proton pump inhibitor.

*Manual Surgical Sciences, Minneapolis, MN, USA.

**Esosure, Boynton Beach, FL, USA.

## References

[1] Han HC, Ha FJ, Sanders P (2017). Atrioesophageal fistula: clinical presentation, procedural characteristics, diagnostic investigations, and treatment outcomes.. Circ Arrhythm Electrophysiol..

[2] Nair GM, Nery PB, Redpath CJ, Lam BK, Birnie DH (2014). Atrioesophageal fistula in the era of atrial fibrillation ablation: a review.. Can J Cardiol..

[3] Halbfass P, Pavlov B, Muller P (2017). Progression from esophageal thermal asymptomatic lesion to perforation complicating atrial fibrillation ablation: a single-center registry.. Circ Arrhythm Electrophysiol..

[4] Gupta A, Perera T, Ganesan A (2013). Complications of catheter ablation of atrial fibrillation: a systematic review.. Circ Arrhythm Electrophysiol..

[5] Kapur S, Barbhaiya C, Deneke T, Michaud GF (2017). Esophageal injury and atrioesophageal fistula caused by ablation for atrial fibrillation.. Circulation..

[6] Maenosono R, Oketani N, Ishida S (2012). Effectiveness of esophagus detection by three-dimensional electroanatomical mapping to avoid esophageal injury during ablation of atrial fibrillation.. J Cardiol..

[7] Chavez P, Messerli FH, Casso Dominguez A (2015). Atrioesophageal fistula following ablation procedures for atrial fibrillation: systematic review of case reports.. Open Heart..

[8] Zellerhoff S, Lenze F, Schulz R, Eckardt L (2011). Fatal course of esophageal stenting of an atrioesophageal fistula after atrial fibrillation ablation.. Heart Rhythm..

[9] Singh SM, d’Avila A, Singh SK (2013). Clinical outcomes after repair of left atrial esophageal fistulas occurring after atrial fibrillation ablation procedures.. Heart Rhythm..

[10] Cazavet A, Muscari F, Marachet MA, Leobon B (2010). Successful surgery for atrioesophageal fistula caused by transcatheter ablation of atrial fibrillation.. J Thorac Cardiovasc Surg..

[11] Khandhar S, Nitzschke S, Ad N (2010). Left atrioesophageal fistula following catheter ablation for atrial fibrillation: off-bypass, primary repair using an extrapericardial approach.. J Thorac Cardiovasc Surg..

[12] St. Julien J, Putnam JB, Nesbitt JC, Lambright ES, Petracek MR, Grogan EL (2011). Successful treatment of atrioesophageal fistula by cervical esophageal ligation and decompression.. Ann Thorac Surg..

[13] Bunch TJ, Nelson J, Foley T (2006). Temporary esophageal stenting allows healing of esophageal perforations following atrial fibrillation ablation procedures.. J Cardiovasc Electrophysiol..

[14] Ellis C, Streur M, W Scharf A, Nesbitt J (2012). Successful treatment of esophageal perforation following atrial fibrillation ablation with a fully covered esophageal stent: prevention of atrial-esophageal fistula.. J Innov Cardiac Rhythm Manage..

[15] Eitel C, Rolf S, Zachaus M (2013). Successful nonsurgical treatment of esophagopericardial fistulas after atrial fibrillation catheter ablation: a case series.. Circ Arrhythm Electrophysiol..

[16] Di Biase L, Saenz LC, Burkhardt DJ (2009). Esophageal capsule endoscopy after radiofrequency catheter ablation for atrial fibrillation: documented higher risk of luminal esophageal damage with general anesthesia as compared with conscious sedation.. Circ Arrhythm Electrophysiol..

[17] Martinek M, Bencsik G, Aichinger J (2009). Esophageal damage during radiofrequency ablation of atrial fibrillation: impact of energy settings, lesion sets, and esophageal visualization.. J Cardiovasc Electrophysiol..

[18] Halm U, Gaspar T, Zachäus M (2010). Thermal esophageal lesions after radiofrequency catheter ablation of left atrial arrhythmias.. Am J Gastroenterol..

[19] Sause A, Tutdibi O, Pomsel K (2010). Limiting esophageal temperature in radiofrequency ablation of left atrial tachyarrhythmias results in low incidence of thermal esophageal lesions.. BMC Cardiovasc Disord..

[20] Kumar S, Romero J, Stevenson WG (2017). Impact of lowering irrigation flow rate on atrial lesion formation in thin atrial tissue.. JACC Clin Electrophysiol..

[21] Leshem E, Zilberman I, Tschabrunn CM (2018). High-power and short-duration ablation for pulmonary vein isolation: biophysical characterization.. JACC Clin Electrophysiol..

[22] Daly MG, Melton I, Roper G, Lim G, Crozier IG (2018). High-resolution infrared thermography of esophageal temperature during radiofrequency ablation of atrial fibrillation.. Circ Arrhythm Electrophysiol..

[23] Tschabrunn CM, Silverstein J, Berzin T (2015). Comparison between single- and multi-sensor oesophageal temperature probes during atrial fibrillation ablation: thermodynamic characteristics.. EP Europace..

[24] Circa Scientific. Circa S-Cath hot and cold esophageal temperature monitoring system.

[25] Mateos JC, Mateos EI, Pena TG (2015). Simplified method for esophagus protection during radiofrequency catheter ablation of atrial fibrillation—prospective study of 704 cases.. Rev Bras Cir Cardiovasc..

[26] Koruth JS, Reddy VY, Miller MA (2012). Mechanical esophageal displacement during catheter ablation for atrial fibrillation.. J Cardiovasc Electrophysiol..

[27] Palaniswamy C, Koruth JS, Mittnacht AJ (2017). The extent of mechanical esophageal deviation to avoid esophageal heating during catheter ablation of atrial fibrillation.. JACC Clin Electrophysiol..

[28] Bhardwaj R, Naniwadekar A, Whang W (2018). Esophageal deviation during atrial fibrillation ablation: clinical experience with a dedicated esophageal balloon retractor.. JACC Clin Electrophysiol.

[29] Parikh V, Swarup V, Hantla J (2018). Feasibility, safety, and efficacy of a novel preshaped nitinol esophageal deviator to successfully deflect the esophagus and ablate left atrium without esophageal temperature rise during atrial fibrillation ablation: the DEFLECT GUT study.. Heart Rhythm..

